# Molecular genotyping of adrenocortical carcinoma: a systematic analysis of published literature 2019–2021

**DOI:** 10.1097/CCO.0000000000000799

**Published:** 2021-10-19

**Authors:** Salvatore Grisanti, Deborah Cosentini, Sandra Sigala, Alfredo Berruti

**Affiliations:** a Medical Oncology Unit, Department of Medical and Surgical Specialties, Radiological Sciences, and Public Health, University of Brescia, ASST Spedali Civili; b Section of Pharmacology, Department of Molecular and Translational Medicine, University of Brescia, Brescia, Italy

**Keywords:** adrenocortical carcinoma, DNA damage repair, genomic landscape, immunotherapy, methylation

## Abstract

**Purpose of review:**

comprehensive molecular characterization of adrenocortical carcinoma (ACC) through next-generation sequencing and bioinformatics analyses is expanding the number of targets with potential prognostic and therapeutic value. We performed a critical review of recent published literature on genotyping of ACC.

**Recent findings:**

423 studies were published between 2019 and 2021. After manual curation we summarized selected evidence in two thematic areas: germline deoxyribonucleic acid (DNA) variations, genomic alterations and prognosis.

**Summary:**

the evolving genomic landscape of ACC requires target validation in terms of prognostic and predictive value within scientific consortia. Although the existing multiple driver genes are difficult targets in the perspective of precision oncology, alterations in DNA damage repair genes or in promoter hypermethylation could open new venues for repurposing of existing drugs in ACC.

## INTRODUCTION

Adult adrenocortical carcinoma (ACC) is a rare neoplasm with a worldwide reported incidence of 0.7–1.0 new cases per million people/year [1]. Because of the rarity of this disease, the expected trends of incidence and mortality are difficult to define and access to this information relies only on collection of data within ACC-specific registries. Prognosis of ACC is variable but more than 60% of patients are diagnosed in stage III and IV with a 5-year survival of <50% and <15%, respectively. For these patients, the proposed algorithm of treatment includes systemic treatment with mitotane, platinum-based chemotherapy (e.g. EDP-M schedule) and locoregional strategies including surgery in cases with a residual disease of approximately 10% of initial volume [2,3]. However, progression of advanced disease occurs almost invariably after less than 18 months and there are no defined second and following lines of treatment. In the past 20 years, the therapeutic scenario has not changed substantially and neither molecular target therapies nor immunotherapy with immune-checkpoint inhibitors (ICI) have gained significant results [4]. 

**Box 1:**
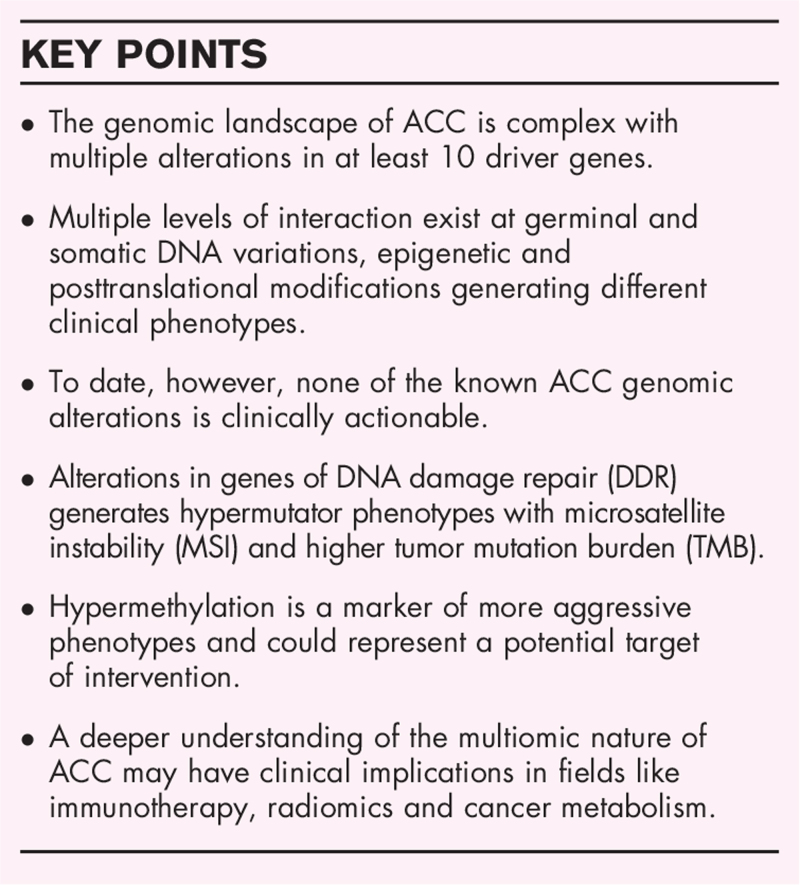
no caption available

Cancer genotyping represents the foundation for precision medicine that holds the promise to illuminate the molecular pathogenesis of each cancer type and to enable clinicians to identify and treat each single patient or molecularly defined groups of patients.

The molecular pathogenesis of ACC has evolved in the last twenty years from description of single chromosomal alterations and identification of single-gene deregulations toward a more comprehensive view with simultaneous analysis of multiple alterations at levels of the deoxyribonucleic acid (DAN) (germinal, somatic, epigenetic), RNA (mRNA, miRNA) and proteins [5–7]. This step forward has been possible essentialy thanks to a wider availability of massive parallel sequencing (i.e. next-generation sequencing [NGS]) technology and to the advancement of bioinformatic analyses across big international scientific consortia [8].

The amount of scientific publications dealing with precision medicine in ACC has been steadily increasing in the last ten years. In this review, we describe the results of a systematic analysis of literature published in the years 2019–2021 concerning genotyping of ACC.

## METHODOLOGY OF LITERATURE ANALYSIS

**FIGURE 1:**
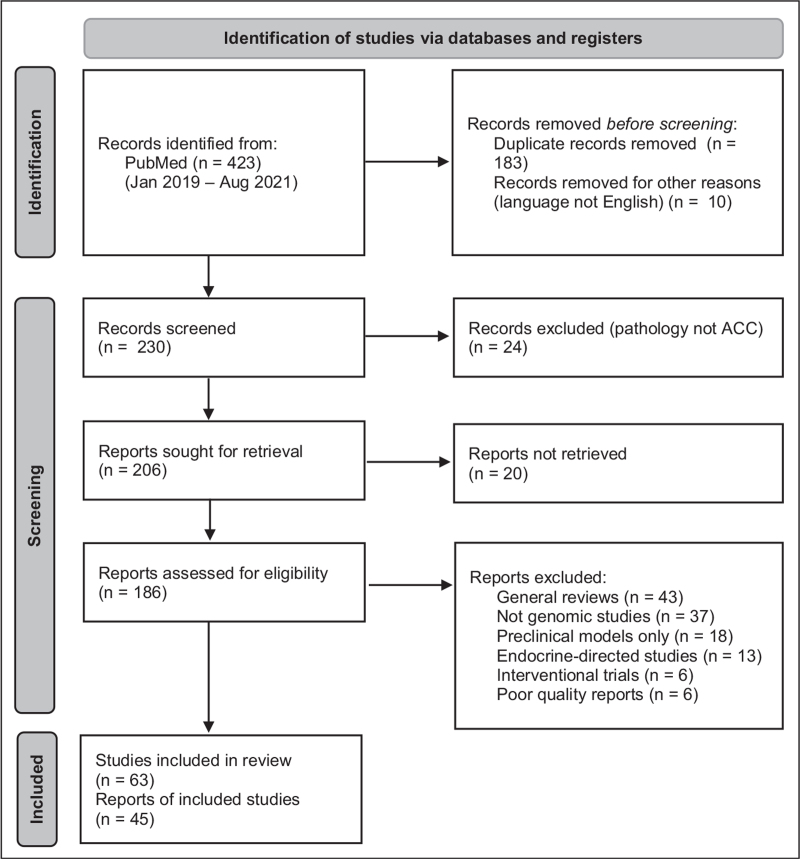
PRISMA flow diagram for systematic review of literature of genotyping of ACC (2019–2021). ACC, adrenocortical carcinoma.

Analysis of literature was conducted following the 2020 Preferred Reporting Items for Systematic Reviews and Meta-analyses (PRISMA) reporting guideline [9]. The National Institutes of Health/National Center for Biotechnology Information (NCBI) PubMed database was queried to identify all original studies published in English between 2019 and 2021 (last accessed August 15, 2021) under the following search terms: ‘adrenocortical carcinoma’ [and] ‘genomic’, ‘genetic’, ‘gene’, ‘germinal’, ‘germline’, ‘epigenetic’, ‘methylation’. Results of search were curated manually. A flow diagram of systematic analysis of literature is provided in Fig. 1. This review is structured in a synthetic background of the current genomic landscape of ACC followed by results of the literature search.

## SUMMARY OF CURRENT GENOMIC LANDSCAPE OF ADRENOCORTICAL CARCINOMA

The genomic landscape of ACC is complex and differentiates this neoplasm from other cancer types with only one or few predominant gene alterations. Comparative studies of pathobiology of ACC revealed that genomic alterations affect genes and molecular pathways that are involved in normal adrenal cortex physiology [10]. In 2019, two separate works reviewed and summarized these genes/pathways that are functional in normal organogenesis and homeostasis of the adrenal cortex and can be detected across processes of benign (adrenal adenoma, ACA) or malignant (ACC) transformation [11^▪^,^12^] of the adrenal cortex.

The current knowledge of genomic landscape of ACC is the cumulative result of both single-gene analyses and multiomic studies from cooperative consortia [5–7].

A simplified summary of the genomic landscape of ACC in essential nodes can be outlined as follows:

(1) *Chromosomal copy number alterations (CNAs) (aneuploidy):* ACC is frequently hypodiploid compared with other cancer types. However, copy number gains and losses can occur in up to 60% of cases (noisy pattern in the TCGA). This unstable pattern is often associated with whole genome doubling (WGD) that is a marker of poor prognosis and is related to alteration of the telomeres length regulation machinery [7].
(2) *Germinal gene mutations:* ACC can arise in the context of cancer-predisposing syndromes (Li-Fraumeni [TP53], Lynch [NMR genes], Beckwith-Wiedemann [CDKN1C, H19, IGF2, KCNQ1OT1], Carney complex [PRKAR1A], Multiple Endocrine Neoplasia type 1 [MEN1] syndromes) with hereditable genetic lesions of the germinal line in approximately 10% and 50–80% of adult and pediatric cases, respectively [13–15]. Other rarer germinal variants potentially predisposing to ACC have been described in succinate dehydrogenase (SDHx) genes [16] and in *Armadillo*-containing repeat protein 5 (ARMC5) gene [17]. In the majority of adult cases, however, ACC is diagnosed as a sporadic cancer with acquired genomic alterations of the somatic DNA.
(3) *Somatic gene mutations and corresponding functional pathways:* recurring mutations (frequency >10% indicated in square brackets) of candidate driver genes have been detected in genes involved in cell cycle (TP53 [21%], CDKN2A [15%], RB1, CDK4, CCNE1), WNT/beta-catenin signaling (CTNNB1 [16%], ZNRF3 [19%]), chromatin remodeling (MEN1, DAXX), telomere maintenance (TERT [14%], TERF2), protein kinase cAMP-dependent regulatory type I alpha gene (cAMP/PKA signaling) (PRKAR1A [11%]), DNA transcription (MED12) and RNA translation (RPL22) [5–7]. Alteration of the Insuline-Like Growth Factor-2 (IGF2) gene is a hallmark of ACC (loss of heterozigosity in 90% of cases) but overexpression of the corresponding insulin-like growth factor-2/receptor-1 (IGF2/IGF1R) axis seems not to be a driver pathway in ACC as demonstrated by failure of clinical intervention with the anti-IGF2/IGF1R linsitinib [18]. Collectively, the two most frequenly altered pathways in ACC pathobiology are the p53/Rb1 cell cycle pathway and the Wnt/beta-catenin pathway (33–45% and 41% of cases, respectively) [7,11^▪^].
(4) *DNA Damage Repair (DDR) genes:* in the TCGA pan-cancer study, >80% of ACC samples displayed at least one DDR gene alteration including genes involved in: Mismatch Repair (MMR): MLH1-3, MSH2-6, PMS2; Homologous Recombination (HR): TP53BP1, BRCA1-2, BRIP1, RAD51, TOP3A; Damage Sensor (DS): ATM, ATR, CHEK2; Translesion Synthesis (TS): REV3L; Base Excision Repair (BER): POLB; other minor DDR gene alterations involve Direct Repair (ALKBH3, MGMT), Fanconi Anemia (FANCA, FANCD2) and Nonhomologous End Joining (LIG4, XRCC4, XRCC6) [19]. Many of these gene alterations are found at level of both germinal and somatic DNA. In particular, germinal MMR gene alterations observed in familial ACC cases identify familial ACC as a Lynch syndrome (LS)-associated cancer [20].
(5) *Microsatellite instability (MSI):* defects of the MMR system cause microsatellite instability (MSI) that is both prognostic and predictive of response to therapy in many cancer types including colorectal and endometrial cancers. In a pan-cancer re-analysis of TCGA data, Bonneville *et al.* found a MSI-high (MSI-H) phenotype in 4.3% of ACC cases placing ACC as the fifth neoplasm with the highest MSI-H rate among 39 different cancers. The MSI-H phenotype was restricted to cases with a high somatic tumor mutation burden (1157 vs 217 mutations in MSI-H vs MSS ACC, p 0.01) [21].
(6) *Tumor mutation burden (TMB):* in the TCGA-ACC study, the median somatic mutation density was 0.9 mutation/Mb (range 0.2–14.0 mutations/Mb) [7]. In a pan-cancer analysis, ACC had a median TMB less than 5 mutations/Mb and less than 10% of cases had a TMB >10 mutations/Mb. Therefore, despite all of the above considerations, ACC is placed among tumors with the lowest TMB [22].
(7) *Epigenetic changes:* since 2012 at least 7 studies identified DNA methylation as an important mechanism of epigenetic control of gene expression in ACC. Both hypomethylation and hypermethylation of promoter regions can occur at a higher frequency in ACC compared to ACA [23,24]. In the European Network for the Study of Adrenal Tumors (ENSAT) and TCGA-ACC studies, analysis of the hypermethylation at CpG-rich islands defined three phenotypes of methylation (CpG island methylator phenotype –CIMP- high, intermediate and low) that showed a significant prognostic value. In particular, in the CIMP-high profile cases segregated with higher proliferative index and associated with worse prognosis [5,7]. A comprehensive review by Ettaieb *et al.* on the role of epigenetic alterations in ACC and their potential role as prognostic factors and therapeutic targets have been recently published [25].

For the purposes of the present review, we do not enter in greater detail of the ACC genomic landscape but for further reading we recommend three excellent reviews published in the last 3 years [11^▪^,26^▪^,^27^].

## SYSTEMATIC ANALYSIS OF LITERATURE 2019–2021 OF MOLECULAR GENOTYPING OF ADRENOCORTICAL CARCINOMA

Our literature search retrieved a total of 423 publications. After manual curation, duplicate records and works not dealing with adrenal tumors were excluded. Selected reviews were included if, in the opinion of authors, they added significant insight in the field.

We organized published works in two wide thematic areas of interest.

### Germline DNA alterations in adrenocortical carcinoma

The interest for germline alterations in ACC is rising and the burden of new variants are expanding not only in familial syndromic cases but also in sporadic ACC patients. This renewed interest is linked to the potential therapeutic implications of germline mutations and to their clonal nature that renders them ideal candidate as predictive factors [28]. Excluding reviews, 21 original studies were retrieved within the predefined time frame. A summary is provided in Table 1. Nine studies were retrospective analyses of known germinal variants such as TP53 [29,30] and MEN1 [31] in pediatric and adult patients. In one study variants of Epidermal Growth Factor Receptor (EGFR) were identified at higher incidence in children and young adults ACC [32]. Polymorphisms of selected genes could have influence on ACC incidence (retinoic acid pathway genes) [33], ACC tumorigenesis (phosphodiesterases genes) [34] or response to mitotane treatment (P450 cytochrome genes) [35]. Twelve single case reports expanded the catalog of single or multiple variants described in ACC. For example, a CHEK2 germline variant has been identified for the first time in ACC [36] and the presence of a double alteration (MSH2 and RET) has been identified in an adult patient without MEN2 syndrome [37]. Landwehr *et al.* identified a MUTYH variant in an ACC patient with high TMB from whom a new cell line has been derived. Germline MUTYH mutations occur in the context of DDR gene alterations. MUTYH mutations identify MUTYH-associated poliposis (MAP) of the colon and have been described also in ACC. Therefore, the importance of this finding resides in the availability of a preclinical model with a known alteration of the DNA repair machinery and a high TMB [38]. Nine reviews (not cited) were published focusing specifically on germline alterations in benign (eg, primary aldosteronism, ACAs) or malignant adrenal tumors and in pediatric and adult patients with or without familial genetic syndromes [39–49].

**Table 1:** Summary of 2019–2021 published original studies of germline DNA alterations in adrenocortical tumors in chronological order

Year/Author[Reference]	Germinal Mutation	Type of publication	Significance
2018/Xie [36]	CHEK2	Case report	First description of a CHEK2 germinal variant in ACC
2019/Wang [31]	MEN1	Retrospective monocentric series	Report of MEN1 in 1/68 family (2/121 individuals). In this family 2 cases of ACC were diagnosed (prevalence 1.5%)
2019/Nicolson [39]	WES of somatic and germinal DNA	Case report	Different mutational profile of syndromic (case report) vs sporadic ACC (control series)
2019/Mc Cabe [40]	WES of somatic and germinal DNA	Case report	Alteration of MSH2, TP53, RB1, PTEN resulting in a signature of MRP and MSI: implication for immunotherapy.
2019/Ferreira [30]	TP53-R337H	Retrospective trial	Description of the clinical spectrum of Li-Fraumeni syndrome in Brazilian carriers of TP53-H337H mutations
2019/Kaur [41]	MSH6	Case report	Patient with oncocytic ACC as unique manifestation of a familial Lynch syndrome
2019/Tang [42]	TP53	Case report	Patient with neuroblastoma and ACC
2020/Altieri [35]	CYP2W1∗6 &CYP2B6∗6 polymorphisms	Retrospective multicenter ENSAT trial	Effect of germinal polymorphisms of CYP2 enzymes on Mitotane treatment in ACC: CYP2W1∗6 polymorphism is associated with lower probability to achieve terapeutic range compared to CYP2B6∗6
2020/Gagnon [43]	APC	Case report	Demonstration of an APC VUS in the transition adenoma-ACC in an adult patient
2020/Bondy [44]	TP53	Case report	Case of adult onset of Li-Fraumeni syndrome with 3 different neoplasms: breast cancer, ACC, pleomorphic sarcoma.
2020/Surakhy [33]	Retinoic Acid (RA) polymorphisms	Retrospective trial	Polymorphism in the RA pathway has influence on incidence of ACC
2020/Raygada [37]	MSH2 & RET	Case report	Description of double germinal mutations of MSH2 and RET in an adult patient with ACC without MEN2
2020/Suda [45]	Fumarate Hydratase	Case report	Association of fumarate hydratase-dependent cardiac myxoma and ACA
2020/Pinto [34]	Phosphodiesterases genes (PDEs)	Retrospective trial	Inactivating variants of PDEs are found in 24% of pediatric adrenocortical tumors. Possible role of PDEs in the cAMP-signaling pathway and adrenal tumorigenesis
2020/Feitosa [46]	TP53-R337H	Retrospective trial	Prevalence of TP53-R337H within South Brazilian pediatric patients: high prevalence of ACC (3/3)
2021/Takeoka [47]	TP53	Case report	Description of an ACC pediatric case in a Li-Fraumeni syndrome that led to screening of another younger brother with diagnosis of sarcoma
2021/Domenech [48]	MSH2, MSH6	Retrospective trial	Prevalence of 3/634 (0.47%) patients with ACC within context of Lynch syndrome
2021/Landwehr [38]	MUTYH	Cell line report	Description of a new MUTYH germline variant in an ACC-derived cell line
2021/Brenna [29]	TP53	Retrospective trial	Pediatric ACC patients carrying germline TP53 mutations have a more favourable outcome than wild-type counterpart
2021/Torres [49]	Ataxia Teleangectasia Mutated (ATM)	Case report	Identification of a pathogenetic variant of ATM in an adult patient with ACC
2021/Akhavanfard [32]	EGFR	Retrospective trial	High incidence of germline EGFR variants in children (4.8%) and young adults (6.2%) with ACC

ACC, adrenocortical carcinoma; ENSAT, european network for the study of adrenal tumors.

### Somatic genomic alterations in adrenocortical carcinoma

A major goal of precision medicine in oncology is to integrate molecular characteristics with known clinical prognostic factors and, thus, to refine risk stratification of patients. In the last three years, the ACC scientific community invested big efforts in translating the bulk of multiomic data in new prognostic classifications or clinical applications.

In the predefined time frame, 29 original works were published and a summary is provided in Table 2[50,51^▪^,52^▪^,^54^–^67^,68^▪^,^69^–^75^].

**Table 2:** Summary of 2019–2021 published original studies of somatic genomic alterations with potential prognostic impact in adrenocortical tumors in chronological order

Year/Author[Reference]	Description of study	Significance
2019/Assié [51^▪^]	ENSAT multicenter comparative study of clinical vs molecular stratification on 364 ACC patients evaluated with multitarget molecular profiling.	Combination of clinical and molecular classifiers better discriminated prognostic groupsin stage I-III ACC. Molecular classifiers had a limited value in stage IV ACC.
2019/Mohan [52^▪^]	Analysis of prognostic value of the methylation status of the *G0/S2* gene.	Hypermethylation of *G0/S2* gene is a surrogate marker of the CpG-rich islands methylation phenotype (CIMP)-high and identifies ACC patients with very poor prognosis. Potentially useful marker in clinical practice.
2019/Xia [53]	Bioinformatic re-analysis of TCGA data to isolate differently expressed genes in ACC progression.	Analysis identified 4 genes associated with ACC progression: TOP2A, TTK, CHEK1, CENPA.
2019/Liang [54]	Analysis of expression of genes involved in epithelial mesenchymal transition (EMT) in ACC.	Two EMT genes (FSCN1 and FOXM1) are overexpressed in ACC and are associated with poor prognosis.
2019/Xiao [55]	Analysis of differently expressed and methylated genes (DEGs and DMGs) in 92 patients with ACC.	Seven genes with different expression and methylation profiles were identified.
2019/Subramanian [56]	TCGA and cBioPortal databases mining analysis to find new biomarkers.	Overexpression of genes involved in cell-cycle and DNA damage was observed in 82% of cases.
2019/Gao [57]	Gene microarray datasets comparing the gene expression profiles between ACC (47 cases) and adrenal adenoma (46 cases)	20 downregulated genes and 51 upregulated genes, which were highly associated with the cell cycle, organelle fission, chromosome segregation, cell division and spindle stability were found in ACC cases. In particular, cycle 80, cyclin B2 and topoisomerase 2-α were found to be associated with ACC development and overall survival.
2019/Bulzico [58]	Assessment of the association among Twist1, fibronectin, vimentin and E-cadherin gene expression in adrenocortical tumor samples.	Significant correlation between mRNA levels of Twist1, fibronectin and vimentin. No association between Twist1 and E-cadherin expression.
2019/Zhu [59]	Analysis of 36 iron metabolism-related (IMR) genes on 77 ACC cases (TCGA) and 128 normal adrenal tissues.	Among 12 IMR differentially expressed genes, ferroportin1 and ceruloplasmin correlated with poor survival and are potentially implicated in modulating immune responses in ACC.
2020/Li [60]	Molecular alterations and clinical relevance of heterogeneous nuclear ribonucleoproteins (hnRNPs) genes were systematically analysed in 33 cancer types based on next-generation sequence data.	Most hnRNPs were associated with worse survival of ACC patients
2020/Creemers [61]	ENSAT multicenter validation study of the IGF2 methylation score.	In univariate but not in multivariate analysis, the IGF2 methylation score significantly predicted development of metastases after surgery for ACC.
2020/Jouinot [62]	ENSAT multicenter, pan-genomic evaluation of intratumor heterogeneity between primary and metastatic ACC.	Driver gene alterations show a higher level of heterogeneity while methylation and chromosomal alterations profiles are more stable and they may serve as prognostic markers.
2020/Knott [63]	Analysis of (TCGA) the expression of 30 genes encoding the γ-aminobutyric acid (GABA) system in TGCA ACC dataset.	Identification of a subset of ACC patients whose tumors expressed a distinct GABA system transcriptome. This correlated with several favorable clinical outcomes.
2020/Xu [64]	Prognostic evaluation of alternative splicing (AS) events analyzed in 92 ACC patients from TCGA database.	Univariate analysis identified 3919 AS events significantly associated with overall survival.
2020/Dos Santos Passaia [65]	Evaluation of the prognostic significance of STMN1 and its therapeutic potential	STMN1 mRNA levels were significantly higher in ACC patients, especially in an advanced stage, and correlated with BUB1B and PINK1 expression.
2020/Pennanen [66]	Evaluate the role of IDH1 and its mutations in adrenocortical tumors	IDH1 R132H immunohistochemical staining correlated with a better prognosis among ACC patients, but did not distinguish between local and metastasized tumors. Paclitaxel reduces the activation of STMN1 and significantly decreases cell migration and invasion in ACC cell lines.
2020/Li [67]	Bioinformatic re-analysis of transcriptome and clinical TCGA data to isolate and characterize hub-genes of the ACC tumor microenvironment (TME)	A list of 18 hub TME-related genes was identified with poor prognostic value.
2020/Fojo [68^▪^]	Pan-genomic analysis of 42 primary and corresponding 42 metastatic ACCs to search for genes that predispose to disease progression.	Mutational and expression profiles are similar in primary and metastatic ACCs and cannot account for different clinical behaviours.
2021/Yan [70]	Construction of a bioinformatic and machine-learning-based weighted gene co-expression network (WGCNA) model to identify gene-models with potential prognostic value in ACC.	Identification of a multigene model and 6 biomarkers with prognostic value in ACC
2021/Yang [71]	Two independent datasets derived from ACC samples (TCGA-ACC dataset, GEO-GSE76021 dataset) were analysed in order to find prognostic genes.	NDRG4 and CKS2 gene expression has a prognostic impact and may help in risk stratification of ACC.
2021/Deng [72]	Multidimensional bioinformatics analysis to examine the relationship between NRP genes and prognostic and pathological features, tumour mutational burden, microsatellite instability, and immunological features based on public databases and find the potential prognostic value of neuropilins (NRPs).	Low NRP1 expression in ACC was associated with poor prognosis. NRP1 and NRP2 were associated with TMB and MSI.
2021/Shen [73]	Identify the significance of m6A RNA methylation regulators in ACC and construct a m6A based signature to predict the prognosis of ACC patients.	The m6A based signature was an independent prognostic factor for ACC patients.
2021/Fu [74]	Assess the relationships between N6-methyladenosine (m6A)-related genes and ACC through TGCA and GTEx databases.	The expression of m6A-related genes could be used as an independent prognostic factor in ACC
2021/Xu [75]	Analysis of prognostic value of genes implicated in N6-methyladenosine (m6A) RNA methylation in 77 ACC cases from TCGA.	A gene signature built on 3 DEGs genes and on 5 m6A genes identified TNM stage differences in ACC cases and was prognostic of overall survival.

ACC, adrenocortical carcinoma; ENSAT, european network for the study of adrenal tumors.

We concentrate here on a few of them of special interest and potential clinical applications.

In 2018, Lippert *et al.* published results of a genomic analysis of 107 ACC patients. This study demonstrated that genomic-based prognostic stratification improved clinical prognostic models like the mGRAS score and ENSAT stage and that this analytical result could be obtained from standard formalin-fixed, paraffin-embedded tumor tissue [50]. A larger study was published by the ENSAT cooperative group in 2019 [51^▪^] with the experimental hypothesis that molecular stratification could be superior to known clinical prognostic factors. In this retrospective study, TCGA-derived targeted molecular classifiers were used to stratify 364 ACC patients. Results showed that molecular classification was an independent marker of recurrence in stage I-III ACC but had a limited value in stage IV ACC. Again combination of molecular and clinical factors provided the best prognostic model.

Epigenetic alterations, methylation in particular, have an important role in the genomic landscape of ACC as cited above. Researchers from the Michigan University investigated the methylation status of the G0/S2 gene. They found that hypermethylation of the G0/S2 gene is a marker of overall hypermethylation phenotype in ACC (CIMP-high) and, when combined with the validated marker BUB1B-PINK1, identifies a subgroup of patients with rapidly fatal disease. Furthermore, analysis of methylation of single G0/S2 gene is affordable and it could become an useful marker of hypermethylation in clinical practice [52^▪^]. Another study dealing with prognostic impact of methylation has been published by Creemers *et al.*[63]. In this ENSAT study, the methylation status of IGF2 promoter regions was validated along with other clinico-pathological factors. The IGF2 methylation score predicted development of metastases after surgery in univariate analysis but was inferior to the Weiss score in multivariate analysis.

Two studies focused on molecular heterogeneity between primary and recurrent/metastatic ACC. In one study, Jouinot *et al.* found a higher level of intratumor heterogeneity in driver genes that are considered founder core alterations of the tumor while relatively more stable profiles of methylation and chromosomal alterations [62]. In line with this result, Fojo *et al.* did not find significant differences in driver genes mutation and expression profiles between primary and metastatic ACCs [68^▪^]. This result is in partial contrast with a previous report by Gara *et al.* who found a higher mutation rate in metastatic vs primary ACCs and an overlap of 37–57% in mutated genes among different sites from the same patient [69].

Other studies published between 2019 and 2021 are summarized in Table 2. Many of these studies are bioinformatic re-analyses of existing datasets (e.g. TCGA), whereas others concentrate on novel genes/pathways analysis but lack validation. It is beyond the scopes of this review to discuss in detail each single study.

At the time of writing the present manuscript, a single center study from the University of Colorado has been published. In this work, the authors obtained from Foundation Medicine Inc. (FMI) genomic and partial demographic data of 364 ACC patients, whereas clinical data were not provided. This work represents to date the largest analysis of somatic genomic alterations in ACC by a FDA-approved, commercially available test. The analysis expanded further the catalog of somatic gene alterations and highlighted that patients with a high tumor mutation rate have an unprecedently reported high incidence of alterations in the MMR genes (>13% vs median 3% in previous series) [76]. Despite the quantitative relevance of the dataset, this study is unlikely to have a clinical impact as correlations with clinico-pathological data are not reported (we were not able to retrieve demographic information from on-line supplementary material of the study).

Twenty-five studies have been published between 2019 and 2021 dealing with genomic alterations and potential therapeutic applications. These studies included analyses of prediction to chemotherapy agents, target therapies and currently available and future immunotherapies.

## CONCLUSION

The rising number of publications concerning the multifaceted molecular alterations of ACC underlines the strong scientific interest in this complex and difficult orphan disease. New emerging evolutions of the current landscape of ACC are linking classical genomics with other fields such as immunology [77,78], pharmacology [79] and cancer metabolism [80].

From the clinical point of view, all together the above cited data can be relevant for prognostic stratification, prediction of sensitivity to chemotherapy and/or immunotherapy with ICIs and can be useful for designing future trials in ACC [81,82]. On the other hand, as noted by Fojo *et al.*, despite the large evidence of genomic data accumulated on approximately 200 ACC patients, to date none of the molecular alterations in ACC is therapeutically actionable [68^▪^].

## Acknowledgements

*The authors have no conflicts of interest to disclose.*

*The authors would like to aknowledge the work of all researchers involved in ACC that is not directly cited in this review for space limitations.*

### Financial support and sponsorship

*None.*

### Conflicts of interest

*There are no conflicts of interest.*

## REFERENCES AND RECOMMENDED READING

Papers of particular interest, published within the annual period of review, have been highlighted as:

▪ of special interest
▪▪ of outstanding interest
